# Prevalence of leisure-time sedentary behaviour and sociodemographic correlates: a cross-sectional study in Spanish adults

**DOI:** 10.1186/1471-2458-14-972

**Published:** 2014-09-19

**Authors:** Ricardo Macías, María Garrido-Muñoz, Carlos M Tejero-González, Alejandro Lucia, Enrique López-Adán, Gabriel Rodríguez-Romo

**Affiliations:** Facultad de Ciencias de la Actividad Física y del Deporte, Universidad Europea de Madrid, Madrid, Spain; Facultad de Formación de Profesorado y Educación, Universidad Autónoma de Madrid, Madrid, Spain; Universidad Europea de Madrid and Instituto de Investigación 12 de Octubre (i + 12), Madrid, Spain; Facultad de Ciencias de la Actividad Física y del Deporte - INEF, Universidad Politécnica de Madrid, Madrid, Spain

**Keywords:** Physical activity, Sedentarism, Leisure-time, Adults, Madrid

## Abstract

**Background:**

Being physically inactive has been linked to a higher mortality and poorer quality of life. This cross-sectional study examines the prevalence of leisure-time sedentary behaviour in a population of Spanish adults and its correlates with several sociodemographic variables.

**Methods:**

Data were collected from 1,330 subjects living in Madrid (age: 18-65 years, 51.6% women) by telephone interview. The sampling error was ±2.7% for a 95.5% confidence level. Leisure-time sedentary behaviour was assessed using the Global Physical Activity Questionnaire (version 2). Further factors examined were: country of birth, sex, age, civil state, education level, employment and economic status and physical activity of parents.

**Results:**

76.3% of the subjects interviewed reported a mostly sedentary leisure-time lifestyle. The remaining subjects (23.7%) reported a moderate to high level of physical activity, meeting minimum recommendations. Logistic regression adjusted for all variables identified the following population subsets as showing a greater risk of sedentary behaviour: women (odds ratio (OR) = 2.14; 95% confidence interval (CI): 1.64, 2.79), participants aged 41-50 years (OR = 1.64; 95%CI:1.05, 2.51), those with a middle economic status (OR = 1.48; 95% CI: 1.04, 2.10) or not providing information about their income (OR = 1.97; 95% CI: 1.05, 3.67), and those whose father (OR = 1.53; 95% CI: 1.13, 2.07) and/or mother (OR = 1.41; 95% CI: 1.01, 1.97) were never physically active during leisure-time.

**Conclusions:**

The high prevalence of self-reported sedentary behaviour recorded suggests the need for public health policies targeted at increasing leisure-time physical activity levels. Our data identified several population subsets as priority candidates for possible interventions pursuing this goal.

## Background

Lifestyle has been identified as a determining factor for mortality and morbidity [1, 2] and several studies have shown that physical activity (PA) is protective against both physical and mental illness, and perhaps even against death [3–6]. Conversely, physical inactivity has been linked to higher mortality [6–9] and a poor quality of life [4, 10, 11].

In an effort to stress the health benefits of PA, several institutions have issued guidelines indicating minimum recommended PA levels in terms of its frequency, duration and intensity. The most accepted guidelines have been those jointly drawn up by the Centers for Disease Control and Prevention and the American College of Sports Medicine (ACSM) in 1995 [7]. These recommendations were later updated in 2007 by the ACSM and American Heart Association (AHA) [12]. In general terms, these recommendations suggest the need for at least 30 minutes of moderate-intensity PA five days per week or a minimum of three days of intense PA performed at least 20 minutes each day, or a combination of both. Despite the evident benefits of regular PA, a large proportion of the population undertakes no PA whatsoever or does not meet the minimum recommendations described above [4, 13, 14].

Physical inactivity is a major source of concern in western societies. Population studies conducted in different countries [15] have reported a mean physical inactivity prevalence of 17.7% if considering total PA, that is, if including all domains of PA (work or daily occupation, leisure-time, and transportation, i.e., walking or cycling to get from one place to another). However, if only the leisure-time PA (LTPA) domain is considered, the prevalence of physical inactivity is higher. Thus, last Eurobarometer survey “Sport and Physical Activity” in 2013 [16], found that 59% of Europeans never exercise or practice any type of sport, or only do so seldom. This percentage was still higher in the southern countries of the European Union.

These data point to a clear need to define intervention strategies designed to encourage PA and, especially LTPA. Furthermore, among the different domains of total PA, LTPA (understood as PA or sport unrelated to work or transport [4]), has been positively linked to different health indicators [17–20]. Accordingly, efforts targeted at promoting PA should be in large measure directed at this component of total PA [21, 22].

For designing and implementing interventions to promote LTPA, a first step is to identify which population groups are more sedentary during their leisure-time. Therefore, it is necessary to understand the influence of sociodemographic correlates on physical inactivity during leisure-time. This information would help define targeted strategies for LTPA promotion in risk groups.

The aims of the present study were to determine the prevalence of leisure-time sedentarism in a representative sample of adults living in the Madrid region (Spain) and to examine its possible associations with several sociodemographic correlates and other variables: place of birth, sex, marital status, education level, employment and economic status and parents’ model of PA.

## Methods

### Study design and population

A cross-sectional study was performed in which data were collected in a structured telephone interview. The sample size determined was 1,330 subjects aged 18 to 65 years living in Madrid region. The sampling error was ± 2.7% for a 95.5% confidence level. The sampling procedure was random and stratified such that the sample was proportional to the population structure in terms of sex, age and geographical area of residence. Participants were selected from those living in homes with a landline telephone (88.5% of all homes in 2009) [23]. Home telephone numbers called to perform the interview were randomly selected from the telephone directory of each geographic area until reaching the planned number of interviews. In each home, only one person was interviewed according to age and sex data for each geographical area. All interviews were administered by five specialists between March and June, 2009. Response rate was 28%. Questionnaires were manually completed by the interviewers. Participation was voluntary and confidential, and informed consent was obtained from participants prior to conducting the survey. Ethical approval for this study was obtained from the Ethics Committee of Faculty of Sports Science, European University of Madrid (in year 2008). The study was performed in accordance with the Declaration of Helsinki and was supervised by the Directorate General of Sports of the Community of Madrid (*Dirección General de Deportes de la Comunidad de Madrid*).

### Assessing leisure-time sedentary behaviour

The dependent variable examined in this study was leisure-time sedentarism, defined as in prior studies [15] as a lack of LTPA or a LTPA level below the recommended PA level [12]. To quantify LTPA and thus identify sedentary subjects, we used the Global Physical Activity Questionnaire (GPAQv2) [24, 25], which contains 16 questions. This questionnaire provides information about the intensity (moderate or vigorous), frequency (days in a typical week) and duration (hours and minutes in a typical day) of PA performed across its three domains: [i] occupational (paid or unpaid work, student work, housework or job seeking), [ii] transport-related (walking or cycling) and [iii] leisure-time. In our study, only data related to LTPA were considered.

The GPAQ derives from the International Physical Activity Questionnaire (IPAQ), which has been validated and widely used to assess PA patterns [26, 27]. The GPAQ shows good reliability, positive moderate to strong correlation with the IPAQ and a validity, albeit low, similar to that of other subjective tools designed to evaluate PA patterns including the IPAQ itself [28].

Here we used the Spanish version of the GPAQv2 without modifying the original contents or text of the questionnaire. The GPAQ protocol was strictly adhered to data recompilation and treatment [25].

From the duration (minutes), intensity (moderate, vigorous) and frequency (days per week) of LTPA performed in a typical week, LTPA-related energy expenditure was calculated according to the guidelines of the questionnaire’s data treatment protocol [25]. LTPA was classed as three levels (high, moderate and low) according to the time spent doing LTPA per day in a typical week, the number of days on which this PA was performed and the intensity of this PA [25] (see Table 1). The cut-offs used to establish these three groups were based on PA recommendations [12]. Thus, the participants assigned to the low PA level were defined as “sedentary or insufficiently active during their leisure-time”, i.e., they performed no PA or did not meet the minimum recommendations for PA to have a health benefit. In contrast, those assigned to the moderate and high PA levels were individuals sufficiently active, i.e., those who met or exceeded these recommendations.

**Table 1:** **Criteria used to define leisure-time physical activity (LTPA) levels included in the GPAQ data analysis protocol**

LTPA level		Inclusion criteria
**1**	**High**	• ≥3 days of leisure-time vigorous activity in a typical week amounting to at least 1500 MET-minutes per week of LTPA or
		• ≥7 days of leisure-time moderate and vigorous activity in a typical week amounting to at least 3000 MET-minutes per week of LTPA.
**2**	**Moderate**	Not meeting the criteria for a “high” level of LTPA but fulfilling some of the following three criteria:
		• ≥3 days of leisure-time vigorous activity in a typical week of at least 20 min duration per day or
		• ≥5 days of leisure-time vigorous and moderate activity in a typical week, of at least 30 min duration per day or
		• ≥5 days of leisure-time moderate and vigorous activity in a typical week amounting to at least 600 MET- minutes per week of LTPA.
**3**	**Low**	Not meeting the criteria for a “high” or “moderate” LTPA level

### Sociodemographic and other variables

The following sociodemographic variables (as independent variables) examined were: country of birth (Spain, other), sex, age (four categories: 18-30, 31-40, 41-50, and 51-65 years), education level (primary or less, secondary, university), civil state (single, married, separated/divorced, widowed), and employment (student, employed, unpaid work/housework, unemployed, retired) and economic status. To evaluate the latter variable, participants were asked to choose the category that was more representative of their economic status considering their family or own incomes: high, middle to high, middle, low to middle, or low. There were no established specific cut-off points between categories and thus selection of one or other category was based on the self-perception of participants [29]. Subsequently, in order to have economic categories of a more homogeneous size, they were re-coded into the following three: high/middle to high, middle, and middle to low/low.

Besides the sociodemographic variables indicated, we also obtained information on the PA habits of the subjects’ parents. To this end, participants were asked whether their mother and father, currently (if alive at the time the interview took place) or only in the past (if not alive), performed LTPA “often”, “sometimes/rarely” or “never”.

### Statistical analysis

Bivariate relationships between LTPA categories (low vs. moderate to high) and sociodemographic correlates were analyzed by chi-squared test (*χ*^2^). The strength of association was quantified according to the contingency coefficient (C). The positive or negative nature of the associations detected was identified by corrected typified residuals (*z*) analysis.

The effects of each independent variable on leisure-time sedentary behavior were assessed by logistic regression. Three regression models were calculated: (i) non-adjusted (Model 1); (ii) adjusted for sex and age variables (Model 2); and (iii) adjusted for all variables studied, i.e., country of birth, sex, age, civil state, education level, employment and economic status, and parents’ LTPA (Model 3). Odds ratios (OR) and 95% confidence intervals (95% CI) were determined, using as reference the cohorts or categories which according to the prior *χ*^2^ tests had shown a lower prevalence of individuals with a low LTPA level. All statistical tests were performed using the programme IBM SPSS Statistics20 (IBM Corporation, USA). Significance was set at 95% (p < 0.05).

## Results

The characteristics of the population sample according to LTPA level are shown in Table 2. Of the 1,330 participants, 76.3% (n = 1,015) reported a low level of LTPA and were classed as sedentary during their leisure-time. The remaining 23.7% (n = 315), undertook a moderate to high LTPA level and met minimum PA recommendations. LTPA levels were independent of country of birth (p = 0.293), education level (p = 0.942) or employment status (p = 0.335), yet were associated to the remaining variables examined: sex (p < 0.001), age (p = 0.017), civil state (p = 0.015), economic status (p = 0.048), and paternal (p = 0.008) and maternal PA (p = 0.042). The prevalence of sedentary people (i.e., those with low LTPA level) was lower among the following individuals: those born in Spain (75.9%), men (69.6%), those aged between 18 and 30 years (70.5%), singles (71.8%), those with primary or lower education level (75.7%), students (72.1%), participants with a high or medium-high economic status (70.8%), and those whose father (71.7%) and/or mother (71.8%) frequently perform (or had performed) LTPA.

**Table 2:** **Characteristics of the study sample by leisure-time physical activity (LTPA) level**

		n	%	LTPA level % (z)		χ ^2^(df)	P	C
				Low	Moderate to high			
	**Total**	**1330**	**100**	**76.3**	**23.7**	**—**	**—**	**—**
**Country of birth**	Spain	1218	91.6	75.9 (-1.1)	24.1 (1.1)	1.10 (1)	0.293	0.02
	Other country	112	8.4	80.4 (1.1)	19.6 (-1.1)			
**Sex**	Male	644	48.4	**69.6 (-5.6)**	**30.4 (5.6)**	31.47 (1)	<0.001	0.15
	Female	686	51.6	**82.7 (5.6)**	**17.3 (-5.6)**			
**Age (years)**	18-30	359	27.0	**70.5 (-3.0)**	**29.5 (3.0)**	10.21 (3)	0.017	0.09
	31-40	324	24.4	77.2 (0.4)	22.8 (-0.4)			
	41-50	334	25.1	80.2 (1.9)	19.8 (-1.9)			
	51-65	313	23.5	78.0 (0.8)	22.0 (-0.8)			
**Civil state**	Single	496	37.3	**71.8 (-3.0)**	**28.2 (3.0)**	10.44 (3)	0.015	0.09
	Married	743	55.9	**78.7 (2.3)**	**21.3 (-2.3)**			
	Separated	64	4.8	84.4 (1.6)	15.6 (-1.6)			
	Widow	2.7	2.0	74.1 (-0.3)	25.9 (0.3)			
**Education level**	Primary or less	358	26.9	75.7 (-0.3)	24.3 (0.3)	0.11 (2)	0.942	0.00
	Secondary	481	36.2	76.7 (0.3)	23.3 (-0.3)			
	Further	491	36.9	76.4 (0.0)	23.6 (0.0)			
**Employment status**	Student	129	9.7	72.1 (-1.2)	27.9 (1.2)	4.56 (4)	0.335	0.05
	Employed	768	57.7	78.3 (1.9)	21.7 (-1.9)			
	Housework	163	12.3	73.6 (-0.9)	26.4 (0.9)			
	Unemployed	189	14.2	75.7 (-0.2)	24.3 (0.2)			
	Retired	81	6.1	72.6 (-1.0)	27.4 (1.0)			
**Economic status**	High or middle-high	240	18.0	**70.8 (-2.2)**	**29.2 (2.2)**	7.39 (2)	0.048	0.07
	Middle	680	51.1	77.5 (1.0)	22.5 (-1.0)			
	Low-middle or low	319	24.0	76.2 (-0.1)	23.8 (0.1)			
	No reply	91	6.8	82.4 (1.4)	17.6 (-1.4)			
**LTPA of father**	Often	329	24.7	**71.1 (-2.6)**	**28.9 (2.6)**	9.56 (2)	0.008	0.09
	Sometimes, rarely	92	6.9	72.7 (-1,3)	27.3 (1.3)			
	Never	909	68.3	**78.8 (3.1)**	**21.2 (-3.1)**			
**LTPA of mother**	Often	245	18.4	71.8 (-1.8)	28.2 (1.8)	7.49 (2)	0.042	0.07
	Sometimes, rarely	78	5.9	72.5 (-1.2)	27.5 (1.2)			
	Never	1007	75.7	**77.9 (2.3)**	**22.1 (-2.3)**			

*Abbreviations*: n number of individuals, % percentage of individuals, z typified residuals, χ^2^ value of chi-squared test, df degrees of freedom, P statistical significance probability, C contingency coefficient. In bold: percentages and typified residuals statistically significant.

Logistic regression analyses are presented in Table 3. When using Model 3 (i.e., regression adjusted for all studied variables), the individuals with higher likelihood of being sedentary during leisure-time were the following: women (OR = 2.14; 95% CI: 1.64, 2.79), people aged 41 to 50 years (OR = 1.64; 95% CI:1.05, 2.51), participants with a medium economic status (OR = 1.48; 95% CI: 1.04, 2.10) or who failed to report this indicator (OR = 1.97; 95% CI: 1.05, 3.67) and people whose father (OR = 1.53; 95% CI: 1.13, 2.07) and/or mother (OR = 1.41; 95% CI: 1.01, 1.97) had never performed LTPA. In contrast, people with a housework occupation had a lower possibility of being sedentary in their leisure-time (OR = 0.32; 95% CI: 0.16, 0.63).

**Table 3:** **Logistic regression analyses of the association between leisure-time sedentary behaviour and the studied socio-demographic correlates**

		Model 1			Model 2			Model 3		
		OR	95% CI	P	OR	95% CI	P	OR	95% CI	P
**Country of birth**	Spain	1	*reference*	—	1	*reference*	—	1	*reference*	—
	Other country	1.29	0.79-2.10	0.294	1.37	0.84-2.25	0.204	1.39	0.84-2.89	0.192
**Sex**	Male	1	*reference*	—	1.00	*reference*	—	1.00	*reference*	—
	Female	**2.08**	**1.60-2.70**	<0.001	**2.07**	**1.60-2.69**	<0.001	**2.14**	**1.64-2.79**	<0.001
**Age (years)**	18-30	1	*reference*	—	1	*reference*	—	1	*reference*	—
	31-40	**1.41**	**1.01-2.99**	0.048	**1.45**	**1.02-2.06**	0.035	1.35	0.91-1.99	0.129
	41-50	**1.70**	**1.19-2.41**	0.003	**1.69**	**1.18-2.41**	0.004	**1.64**	**1.05-2.51**	0.027
	51-65	**1.48**	**1.04-2.10**	0.028	**1.48**	**1.04-2.11**	0.029	1.57	0.96-2.58	0.071
**Civil state**	Single	1	*reference*	—	1	*reference*	—	1	*reference*	—
	Married	**1.45**	**1.11-1.89**	0.005	1.25	0.87-1.79	0.215	1.20	0.83-1.73	0.323
	Separated	**2.12**	**1.05-4.28**	0.036	1.85	0.87-3.93	0.110	1.69	0.79-3.64	0.173
	Widow	1.12	0.46-2.71	0.796	0.78	0.29-2.09	0.624	0.76	0.28-2.06	0.597
**Education level**	Primary or less	1	*reference*	—	1	*reference*	**—**	1	*reference*	—
	Secondary	1.05	0.76-1.45	0.820	1.23	0.88-1.73	0.222	1.31	0.92-1.85	0.125
	Further	1.03	0.75-1.42	0.732	1.17	0.84-1.63	0.329	1.35	0.95-1.92	0.090
**Employment status**	Student	1	*reference*	—	1	*reference*	—	1	*reference*	—
	Employed	1.39	0.91-2.12	0.123	1.01	0.62-1.63	0.963	0.92	0.56-1.51	0.768
	Housework	1.08	0.64-1.81	0.771	**0.34**	**0.18-0.67**	0.002	**0.32**	**0.16-0.63**	0.001
	Unemployed	1.20	0.72-2.00	0.475	0.84	0.48-1.48	0.568	0.76	0.43-1.34	0.352
	Retired	0.97	0.52-1.81	0.939	0.45	0.20-1.02	0.058	0.44	0.19-1.01	0.054
**Economic status**	High or middle-high	1	*reference*	—	1	*reference*	—	1	*reference*	—
	Middle	**1.41**	**1.01-1.97**	0.039	**1.43**	**1.02-2.00**	0.037	**1.48**	**1.04-2.10**	0.026
	Low-middle or low	1.31	0.90-1.92	0.155	1.31	0.89-1.93	0.164	1.44	0.95-2.17	0.082
	No reply	**1.93**	**1.05-3.54**	0.034	**1.90**	**1.02-3.51**	0.040	**1.97**	**1.05-3.67**	0.033
**LTPA of father**	Often	1	*reference*	—	1	*reference*	—	1	*reference*	—
	Sometimes, rarely	0.97	0.58-1.62	0.930	0.98	0.58-1.64	0.945	0.91	0.54-1.54	0.748
	Never	**1.50**	**1.13-2.00**	0.005	**1.50**	**1.12-2.02**	0.007	**1.53**	**1.13-2.07**	0.006
**LTPA of mother**	Often	1	*reference*	—	1	*reference*	—	1	*reference*	—
	Sometimes, rarely	0.93	0.53-1.64	0.822	0.93	0.53-1.65	0.823	0.62	0.51-1.64	0.778
	Never	**1.37**	**1.01-1.89**	0.046	**1.36**	**1.01-1.90**	0.048	**1.41**	**1.01-1.97**	0.045

*Abbreviations*: OR odds ratio, CI confidence interval, P probability of statistical significance. Model 1: Non-adjusted logistic regression. Model 2: Logistic regression adjusted for sex and age. Model 3: Logistic regression adjusted for all variables in the Table 3. In bold: OR and CI statistically significant.

## Discussion

The findings of this study indicate that the majority (76.3%) of adults living in Madrid are physically inactive or sedentary during their leisure-time. This proportion is slightly higher than that reported by Meseguer et al. [14]. According to the latter authors, 71.2% of adults from Madrid do not fulfil the minimum LTPA recommendations for health. A slight reduction in LTPA (especially low to moderate intensity) was observed among Madrid’s inhabitants from 1995 to 2008. During this period, the percentage of adults not meeting PA recommendations rose from 71.3% to 72.9% [30].

Anyway, these comparisons should be made with caution and viewed more as a way of putting results into context rather than a precise comparison between them. These studies have used different methods to assess PA and different definitions of sedentarism, limiting the direct comparison of the results. According to European Union data collected in 1997 [31], percentages for leisure-time sedentary behaviour ranged from 43.3% (Sweden) to 87.8% (Portugal). For Spain, this figure was 71.0%, which is close to the prevalence rates described in previous studies for Madrid [14, 30].

If we consider the total PA (i.e., sum of PA undertaken at work or daily occupation, during leisure-time and when travelling to and from places), the prevalence of sedentarism is considerably lower. Several studies have shown that PA performed in domains outside leisure-time is an important contribution to fulfilling PA recommendations [32–35]. Guthold et al. [15] examined total PA data corresponding to 51 countries taking part in the World Health Survey, 2002-2003, and observed a mean physical inactivity prevalence of 17.7% (15.2% men, 19.8% women). These same authors estimated the physical inactivity rate in Spain at 27.5% for men and 32.9% for women. Although still high, this prevalence of sedentarism, defined as not meeting PA recommendations considering total PA, is far from the 71-76% inactivity in leisure-time observed for adults in Madrid both in our study and others [14, 30, 31].

These figures indicate a clear need to specifically encourage LTPA targeted at reducing the prevalence of sedentarism among adults. Specially, since some studies suggest that LTPA, rather than PA performed in other domains (active transport, work or habitual occupation), is positively associated with health indicators such as a reduced obesity and cardiovascular risk, and improved health perception [17–20].

The correlates observed in the present study between the prevalence of leisure-time sedentarism and several sociodemographic variables, are in large measure consistent with the trends described in the literature, except for those related to education level or employment status. In this regard, a recent systematic review [36] identified clear differences in LTPA levels according to socioeconomic position (SEP). The latter was defined by indicators such as income, education level and social class (based on occupational class). Most of the studies reviewed by these authors indicate that subjects with a higher SEP were more physically active during their leisure-time than those with a lower SEP.

Considering only the income indicator, most investigations have also detected a positive correlation of this indicator with LTPA (59% for total LTPA and 75% for vigorous LTPA) [36]. These findings are in line with those of the present study, given that we identified the group of participants with a high or middle to high economic status (based on own or family incomes) as showing a higher prevalence of a moderate to high LTPA level. In addition, these data were further supported by a greater tendency towards sedentarism observed among the participants with a middle economic status and, possibly, among those who failed to provide information about their income level. Indeed, some studies have correlated a lack of a reply to such a question with a low economic status [37, 38].

With regard to the level of education, this indicator has been positively correlated with LTPA [16, 35, 36, 39–42]. A commonly provided explanation is that people with a higher education level are more likely to understand the health benefits of regular PA, translating to higher LTPA levels. However, our results suggest that the likelihood of being sedentary during leisure-time is not related with education level. Although such lack of association is not in accordance with the majority of studies in the field, a recent meta-analysis by Beenackers et al. [36] found that LTPA was not associated with educational level in 27% of the studies reviewed. Further, a study conducted in a representative Spanish Mediterranean cohort showed that LTPA was unaffected by educational status in either gender [43].

When analysing the association between employment status and LTPA levels in our cohort, we found that those subjects whose habitual occupation was housework, were the only ones who had a lower likelihood of being sedentary during leisure-time. These findings are partly in line with those of Khaing et al. [35]. These authors noted that people that dedicated most of their time to housework and retired subjects were the population subsets with the lowest probability of not meeting PA recommendations, considering total PA. As PA was examined across each domain, retired people and students showed the greatest LTPA levels. However, persons doing housework as their main activity, showed a LTPA energy expenditure that was among the lowest compared to the remaining groups, indicating that PA performed in the home was the greatest contributor to the total PA level.

Sex and age are the variables that are most consistently associated with the PA behaviour of adults. Accordingly, men are usually more active during leisure-time than women and age is usually inversely related to LTPA level [16, 42, 44]. The results of the present study also confirm these trends, indicating that men and the younger study participants (18-30 years) are the two subsets of adults in which greater percentages exist of individuals performing a moderate to high level of LTPA. Similarly, women and participants aged 41 to 50 years emerged as the groups with a greater likelihood of being sedentary in their leisure-time. In addition, as in prior studies [14, 45], our results indicate that the difference between men and women becomes more evident as the intensity of LTPA increases (data not shown).

The relationship between marital status and PA established in the literature has been inconsistent [42]. Thus, while some authors have observed no link between these factors [46, 47], others have established that married compared to single persons, are more likely to be physically inactive in their leisure-time [31, 48, 49]. As in these latter studies, our results also indicate that married people, as well as separated/divorced individuals, are more likely to adopt a sedentary behaviour during their leisure-time than singles. It could be that the family, social and work responsibilities of married subjects, are the underlying cause of this difference. However, the relationships we found between marital status and PA were overall weak and disappeared in the more adjusted regression Model 3.

Recent reviews have shown that a parental model of PA can have a huge impact on the PA behaviour of children [50, 51]. Several studies have noted that when mothers and fathers undertake regular PA, their children are more likely to do the same [52–54]. Although we examined an adult population, the results obtained reveal that subjects whose mother and/or father performed or had in the past performed regular PA, formed a group that showed a higher prevalence of high LTPA levels. In contrast, participants who lacked such a model were more likely to show sedentary behaviour during their leisure-time.

The limitations of our study that could have influenced its results are first and foremost its cross-sectional design, which prevents extracting causal relationships among the variables analyzed. The lack of data on comorbities is another limitation, because ACSM recommendations on PA mainly apply to healthy subjects. In addition, we cannot rule out overestimation of PA levels by the study participants, since these were self-reported rather than obtained through objective methods [55]. However, a methodological strength, as well as a novelty, of the present study is the use of GPAQ as a measure instrument in a wide and representative cohort of adult population from the Madrid region. Indeed, this instrument was specifically designed to assess PA patterns in their different domains, and has proven valid and reliable for PA assessment [28].

## Conclusions

The results of our study suggest that three out of every four adults living in Madrid show sedentary behaviour in their leisure-time. This high prevalence of sedentarism identifies a need for public health policies designed to promote LTPA. Our results also identify the following groups of people as more likely to adopt a leisure-time sedentary behaviour than their respective reference groups: those aged 41 to 50 years, individuals with middle and probably, with lower economic status, women, and those who lack a parental LTPA model. This type of information could be useful to decide which population groups would perhaps benefit most from intervention strategies designed to increase the LTPA of Madrid’s inhabitants. On the other hand, more research is needed using objective assessment of PA (e.g., accelerometry). Future strategies aiming at promoting PA should also take into account several important environment-related variables that were not analysed here, such as urban design, transport system or availability of recreation areas and green spaces [56, 57].

## Abbreviations

OR: Odds ratio
PA: Physical activity
ACSM: American College of Sport Medicine
AHA: American Heart Association
LTPA: Leisure time physical activity
GPAQ: Global physical activity questionnaire
IPAQ: International physical activity questionnaire
CI: Confidence interval
SEP: Socioeconomic position.
